# Cross-plane Thermoelectric and Thermionic Transport across Au/*h*-BN/Graphene Heterostructures

**DOI:** 10.1038/s41598-017-12704-w

**Published:** 2017-10-26

**Authors:** Nirakar Poudel, Shi-Jun Liang, David Choi, Bingya Hou, Lang Shen, Haotian Shi, Lay Kee Ang, Li Shi, Stephen Cronin

**Affiliations:** 1 Ming Hsieh Department of Electrical Engineering, University of Southern California, Los Angeles, CA 90089 USA; 2 Engineering Product Development (EPD), Singapore University of Technology and Design (SUTD), Singapore, 487372 Singapore; 3 Department of Mechanical Engineering and Texas Materials Institute, University of Texas, Austin, Texas 78712 USA; 4 Mork Family Department of Chemical Engineering and Materials Science, University of Southern California, Los Angeles, CA 90089 USA; 5 Department of Chemistry, University of Southern California, Los Angeles, CA 90089 USA; 6 Department of Physics and Astronomy, University of Southern California, Los Angeles, CA 90089 USA

## Abstract

The thermoelectric voltage generated at an atomically abrupt interface has not been studied exclusively because of the lack of established measurement tools and techniques. Atomically thin 2D materials provide an excellent platform for studying the thermoelectric transport at these interfaces. Here, we report a novel technique and device structure to probe the thermoelectric transport across Au/*h*-BN/graphene heterostructures. An indium tin oxide (ITO) transparent electrical heater is patterned on top of this heterostructure, enabling Raman spectroscopy and thermometry to be obtained from the graphene top electrode *in situ* under device operating conditions. Here, an AC voltage V(ω) is applied to the ITO heater and the thermoelectric voltage across the Au/*h*-BN/graphene heterostructure is measured at 2ω using a lock-in amplifier. We report the Seebeck coefficient for our thermoelectric structure to be −215 μV/K. The Au/graphene/*h-*BN heterostructures enable us to explore thermoelectric and thermal transport on nanometer length scales in a regime of extremely short length scales. The thermoelectric voltage generated at the graphene/*h*-BN interface is due to thermionic emission rather than bulk diffusive transport. As such, this should be thought of as an interfacial Seebeck coefficient rather than a Seebeck coefficient of the constituent materials.

## Introduction

Extensive research on two-dimensional (2D) materials has resulted in a wide range of materials that are available for building van der Waals bonded heterostructures. Vertical stacking of graphene, transition metal dichalcogenides (TMDCs), boron nitride, and phosphorene has been used to engineer various types of electronic and optoelectronic devices including transistors^1–4^, photovoltaics^5,6^, and light emitting diodes^7^, as well as to explore novel physical and chemical phenomena on a fundamental level. These van der Waals bonded heterostructures, in principle, provide a system in which it is possible to control electron and phonon transport independently. 2D materials have recently attracted a lot of attention as potential new TE materials^8,9^. In a recent report, Liang *et al*. proposed a highly efficient solid state thermionic device based on a van der Waals (vDW) heterojunction of graphene/TMDC (e.g., MoS_2_, MoSe_2_, WS_2_, WSe_2_) by exploiting the ultra-low cross-plane conductance of the 2D materials and the thermionic emission over the Schottky barrier between graphene and the 2D materials^8^. In principle, this Schottky barrier can be tuned through gating and chemical doping^8^. These calculations predict that thermionic devices can provide better or comparable power generation and refrigeration efficiency than traditional bulk TE devices^8^. However, these predictions have yet to be verified experimentally.

**Figure 1:**
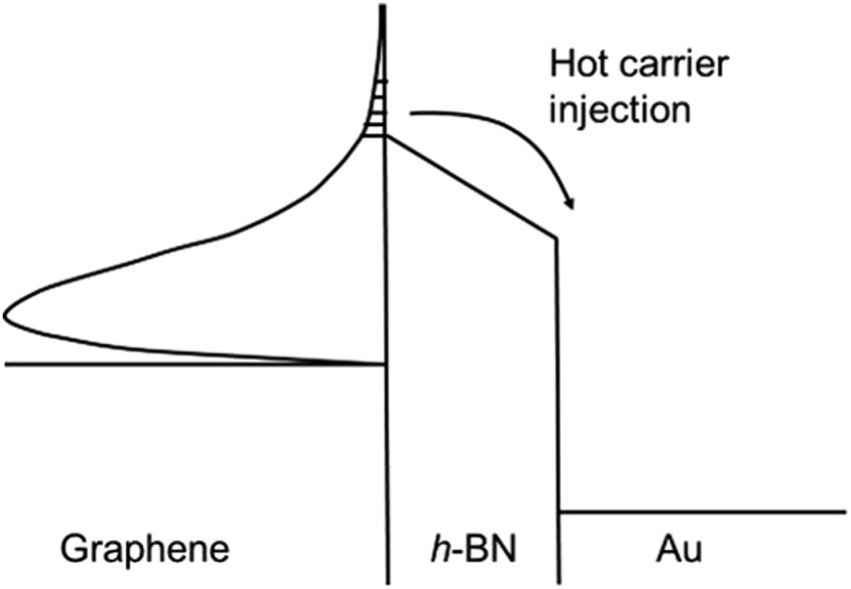
Schematic diagram of the thermionic emission process, illustrated for a graphene/*h*- BN/Au heterostructure.

The efficiency of solid-state thermionic energy conversion has been predicted to exceed that of conventional thermoelectric energy conversion based on bulk Peltier and Seebeck effects, if the thermionic barriers can be properly engineered. Theoretical investigations of this process date back to 1997^8,10–12^. However, there have been relatively few experimental studies on solid-state thermionic energy conversion, mainly because of the difficulty of fabricating interfaces with the appropriate energy barriers, characterizing thermal transport across these interfaces, and separating the bulk thermoelectric properties from the interfacial properties. In thermionic devices, the electrons must flow over the barrier and propagate in the conduction band without scattering in order to have a high efficiency. This requires that the mean-free path *λ* of the electrons in the barrier be longer than the width *L* of the barrier (i.e., *λ* > *L*). This constrains the barrier width *L* to be rather small. On the other hand, the barrier should be thick enough to prevent electrons from quantum mechanically tunneling through the barrier region. The general constraints are *λ* > *L* > *L*_min_, where *L*_min_ is the minimum thickness to prevent the electron from tunneling through the barrier, typically 5–10 nm for most semiconductors. In these devices, electrons traverse relatively thick, low energy barriers (on the order of 2–3*k*_*B*_ *T*). Only electrons in the high energy tail of the Fermi-Dirac distribution will have enough energy to overcome the barrier, resulting in a phenomenon similar to evaporative cooling. A generalized schematic diagram of this process is illustrated in Fig. 1.

In the work presented here, we study the cross-plane thermoelectric and thermionic transport across Au/*h*-BN/graphene heterostructures. This material system is chosen because BN is known to provide a good barrier for electrons (and holes) in graphene, with several previous studies on the graphene/BN/graphene heterostructure system^1,13^. In our structure, we switched the bottom electrode to Au to provide a better heat sink and to enable us to perform Raman thermometry on the top electrode.

## Methods

In the work presented here, we fabricate graphene Au/*h-*BN/graphene heterostructures to explore thermoelectric and thermionic transport across extremely short length scales. There have been several experimental and theoretical studies of thermal transport across graphene/SiO_2_, graphene/Si, and graphene/SiC interfaces^14–23^. Experimental measurements of the thermoelectric transport across a graphene/*h*-BN/graphene heterostructure was previously carried out by Chen *et al*.^13^. In this work, the Seebeck coefficient (*S*) was reported to be −99.3 μV/K and the corresponding power factor (*S* ^2^ *σ*) was 1.51 × 10^−15^ W/K^2^ ^13^. In this previous graphene/BN/graphene thermoelectric measurement, the graphene was used both as a heating element and a temperature monitor. This limited the maximum voltage that could be applied to the heater to ≤2 V, in order to protect the delicate graphene heater from electrical burnout under high currents^13^. Moreover, using graphene as both the heating element and heat sink on the bottom, made it difficult to distinguish the Raman spectra from the top and bottom graphene, which introduces significant inaccuracies in the measurement of the vertical temperature gradient (ΔT) in the active region of the device. In the work presented here, a new device structure was employed to separate the heater and temperature monitor, with the Au/graphene/*h*-BN stack being isolated from the heater by a 50 nm layer of Al_2_O_3_. A 30 nm film of indium tin oxide (ITO) was used as a transparent electrical heater, enabling Raman spectroscopy (and thermometry) to be obtained from the graphene top electrode *in situ* under device operating conditions. As discussed in the works of Vallabhaneni *et al*. and Sullivan *et al*., phonons can be driven out of local equilibrium in suspended graphene under both high and low laser power irradiation^24,25^. The laser powers used in our study (<0.1 mW/µm^2^) is low. Moreover, the heating is provided by an external electrical heater that raises the temperature of different phonons uniformly, while the resulted Raman peak position change is used to extract the temperature rise due to the external heating instead of the constant probe laser heating. As such, the Raman shift provides a reliable measure of the uniform change of the temperatures of different phonon polarizations due to the external heating.

**Figure 2:**
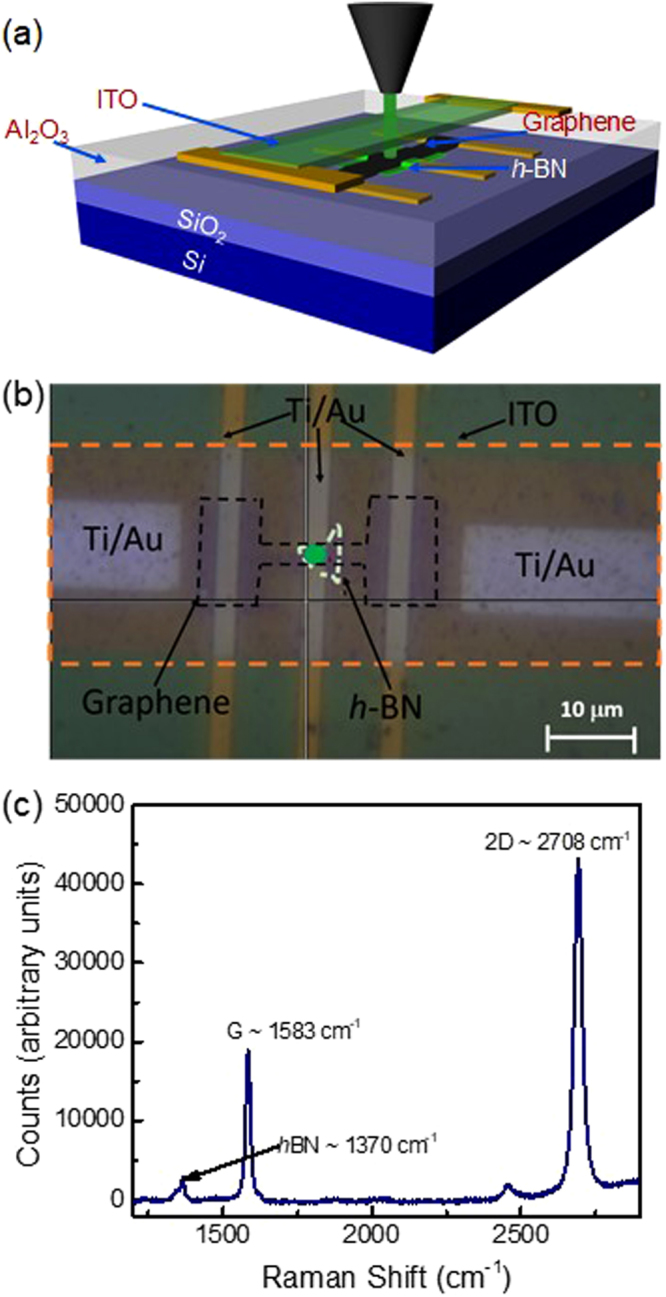
(**a**) Cross-sectional diagram, (**b**) optical microscope image, and (**c**) Raman spectrum of the graphene/h-BN/Au heterostructure with ITO heater.

In the device fabrication process, Ti/Au (5/30 nm) electrodes are deposited on a Si/SiO_2_ substrate using electron beam lithography (EBL) followed by electron beam metal deposition. This bottom gold electrode provides a good heat sink for this thermoelectric device, as described below. A viscoelastic dry transfer process was then used to place an approximately 5 to 10 nm thick *h*-BN flake on top of the center gold electrode, as shown in Fig. 2b ^26^. A wet chemical transfer technique using PMMA as a sacrificial layer was used to transfer graphene grown by chemical vapor deposition (CVD) on copper foil onto the Au/*h*-BN stack. An “I” shaped graphene strip was then patterned, as shown in Fig. 2b, using electron-beam lithography followed by oxygen plasma etching (RF power of 100 W, base pressure of 200 mTorr) for 50 seconds. An insulating layer of Al_2_O_3_ was then deposited on top of the Au/ graphene/*h*-BN sandwich structure using atomic layer deposition. Finally, a 100 μm × 50 μm, 30 nm-thick film of ITO (indium tin oxide) was deposited on top of the oxide via RF sputtering after electron-beam lithography.

## Results and Discussion

**Figure 3:**
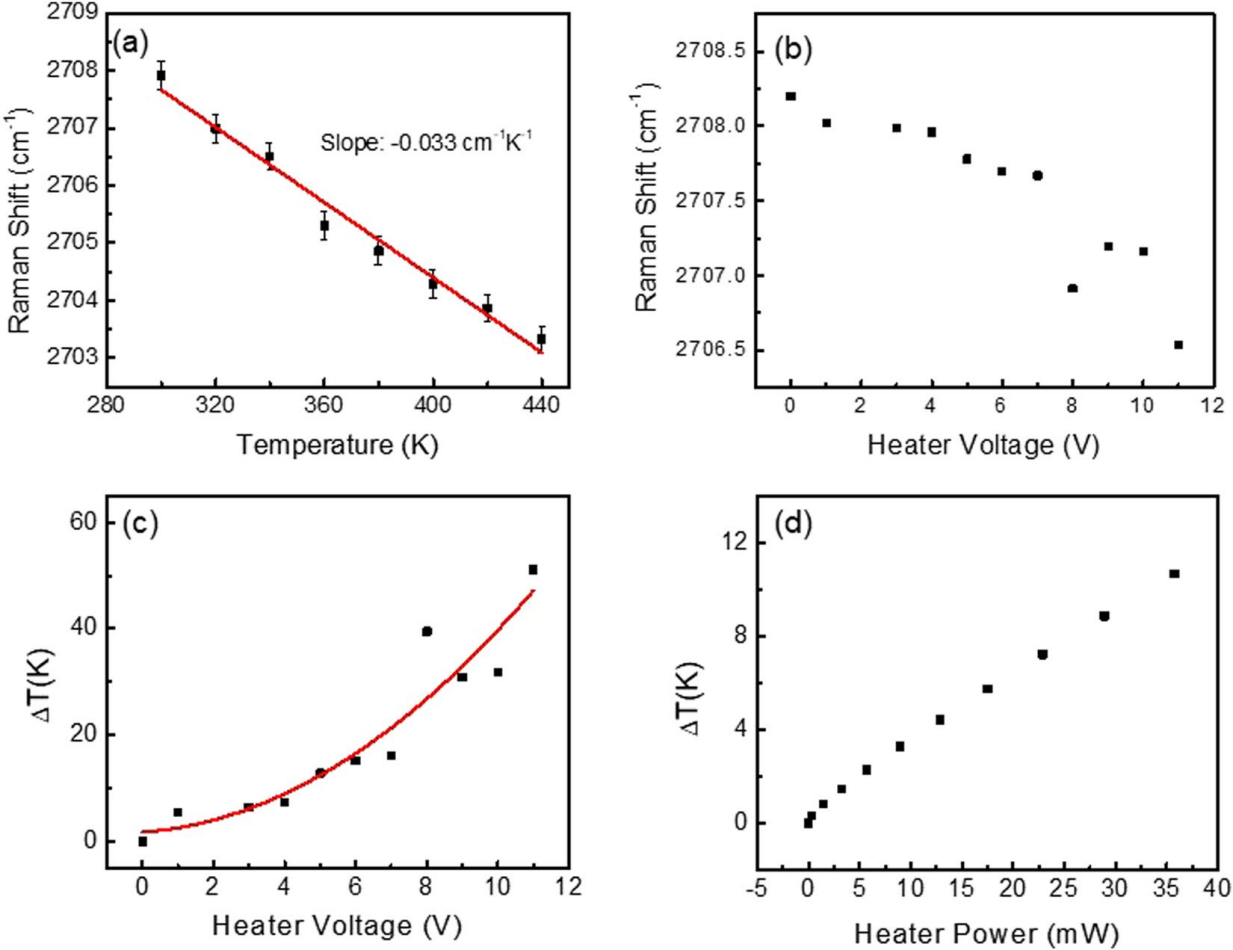
(**a**) Calibration data of the graphene 2D-band Raman shift plotted as a function of temperature obtained in a temperature controlled optical vacuum cryostat. (**b**) Graphene 2Dband Raman shift plotted as a function of the heater voltage. (**c**,**d**) Cross-plane temperature difference plotted as a function of heater voltage and heater power.

After fabrication, the device was placed in a high vacuum, temperature controlled optical cryostat. The Raman spectra of the graphene were acquired using a 532 nm wavelength laser through the transparent ITO heater at various stage temperatures and heater voltages. One such spectrum is plotted in Fig. 2c. Here, distinct peaks can be seen for the G and 2D band Raman modes of the graphene, as well as the optical phonon of *h*-BN at 1370 cm^−1^. The downshifts of the 2D Raman modes of graphene with increasing temperature and heater voltage can be seen in Fig. 3a and b, respectively. This data was then used to determine the temperature of the graphene, and hence the vertical temperature drop (ΔT) across the Au/graphene/*h*-BN heterostructure as a function of the DC heater voltage, as plotted in Fig. 3c. As expected, this ΔT follows a parabolic dependence on the heater voltage and a linear dependence on the heater power (Fig. 2d). In order to verify that the bottom Au electrode remains close to room temperature, we fabricated a separate test structure, in which a monolayer of MoS_2_ was inserted on top of the Au electrode in a Au/MoS_2_/BN/graphene configuration. This device geometry is illustrated in Figure S1 of the Supplement Document. The corresponding temperatures of the graphene and MoS_2_ are plotted as a function of heater voltage in Figure S2, as measured by Raman spectroscopy. This data shows that for every 1 K increase in temperature of graphene, there is a corresponding temperature increase of 0.16 K on average in bottom gold electrode. We use a corrrection factor of ${\rm{\Delta }}T/{T}_{Graphene}$ = 0.84 to account for the slight change in temperature of the bottom gold electrode as a function heating power.

**Figure 4:**
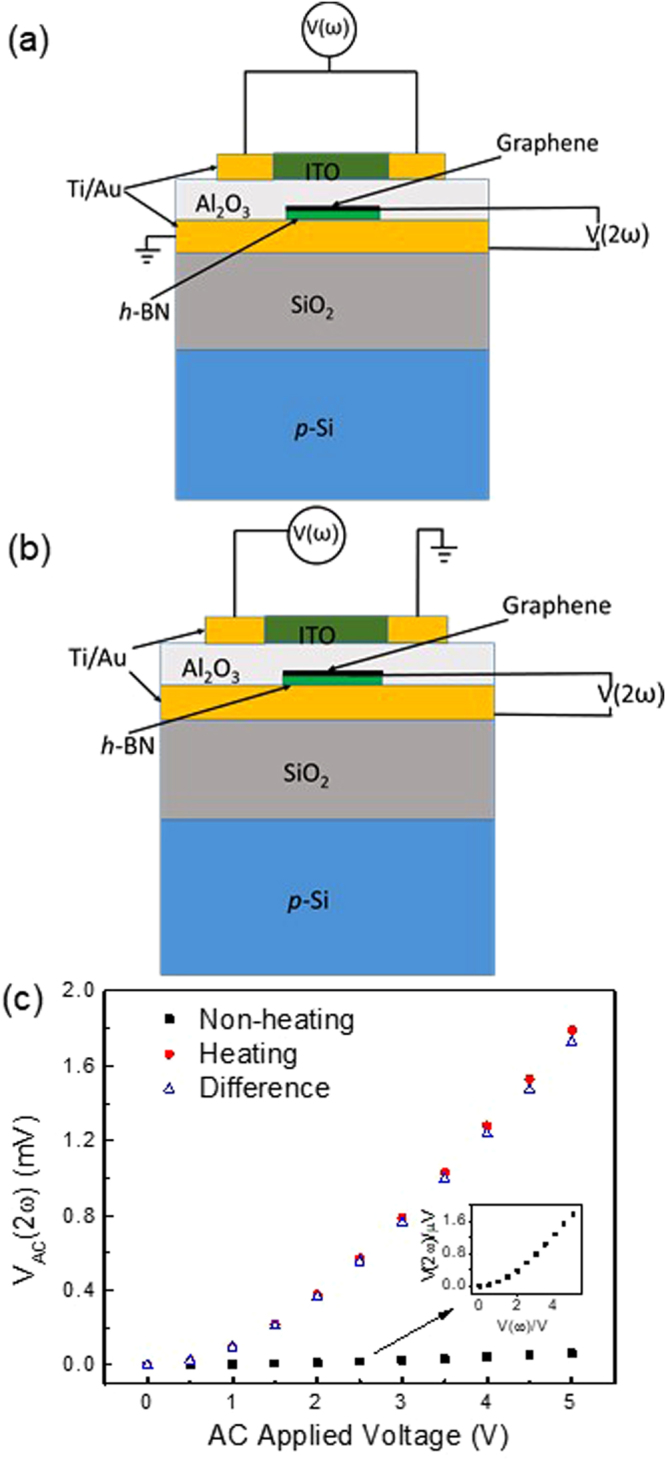
Schematic diagrams of the measurement set up and device geometry for the (**a**) ‘nonheating’ and (**b**) ‘heating’ configurations. (**c**) Cross-plane AC voltage measured between the top graphene contact and bottom Au electrode at 2ω for both heating and non-heating configurations, plotted as a function of the applied AC heater voltage.

**Figure 5:**
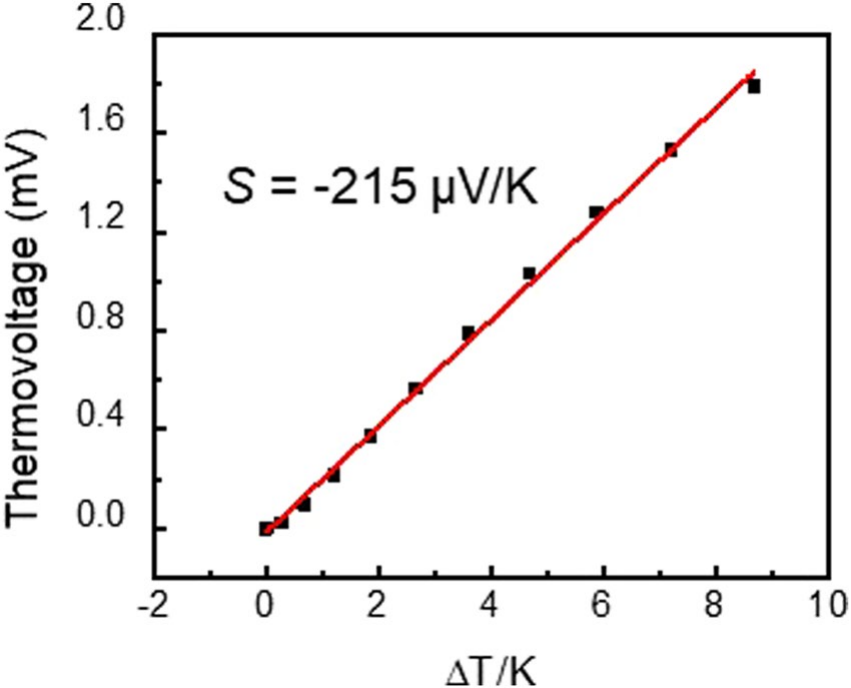
Thermoelectric voltage plotted as a function of the temperature difference across the Au/*h*-BN/graphene heterostructure.

Following this calibration procedure, thermoelectric measurements were performed using an AC lock-in technique. Here, an AC voltage V(ω) was applied to the ITO heater at a frequency of 100 Hz, while the thermoelectric voltage was measured across the Au/*h*-BN/graphene heterostructure at 2ω (i.e., 200 Hz) using a lock-in amplifier. In our previous work, we found that thermovoltage was frequency independent in this range indicating that the thermal time constant of the system is much faster than the AC modulation^13^. The raw data from this measurement is shown in Fig. 4c, which plots the AC thermoelectric voltage measured at 2ω as a function of the AC heater voltage applied. Here, the sample was measured in two different configurations, ‘heating’ and ‘non-heating’, as illustrated in Fig. 4a and b, respectively. In the ‘heating’ configuration, one side of the ITO heater is grounded, enabling heating to occur in the heater when an AC voltage is applied. In the ‘non-heating’ configuration, the AC heater voltage is applied to both sides of the heater, while the underlying Au electrode is grounded. In this configuration, the voltage is applied across the device stack, but does not produce any heating. This non-heating configuration is used to evaluate the effect of second harmonic generation produced across the stack due to non-linearities in the *I*-*V* characteristics of the Au/*h*-BN/graphene device. Whenever an AC voltage is applied to an electronic component with a non-linear *I-V* response, a second harmonic can be generated, which is not thermal in nature. This is particularly important in the case of our 2D sample geometry, since the heater is capacitively coupled to the thermoelectric device. The results shown in Fig. 4c indicate that this second harmonic generation is negligible, and the difference between these two measurements is, in fact, a reliable measure of the thermoelectric voltage generated in these devices. Based on the calibration data shown in Fig. 3c, we can convert the heater voltage in Fig. 4c to temperature difference (ΔT) based on the DC Raman measurements, as plotted in Fig. 5. Here, the thermoelectric voltage exhibits a linear dependence on the temperature gradient, with a Seebeck coefficient of −215 μV/K. The thickness of *h*-BN is estimated to be around 5 to 10 (~3 to 5 nm) layers thick based on the optical contrast of the flake on the 300 nm SiO_2_ substrate. It should be noted that this thermovoltage is created at the graphene/*h*-BN interface rather than in the constituent materials, and originates from thermionic emission mechanism.

**Figure 6:**
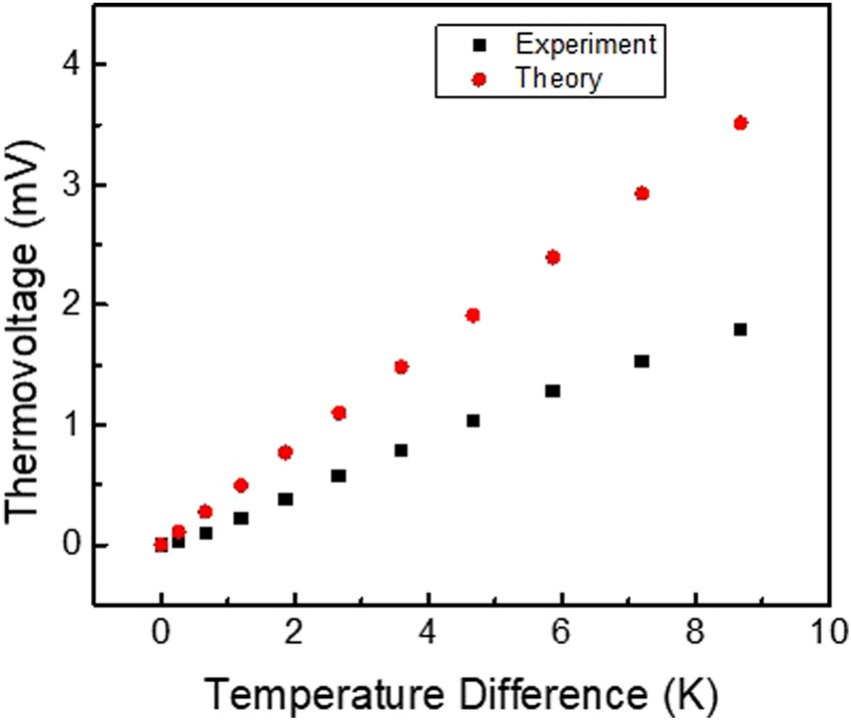
Comparison of theoretical model with experimental measurements for thermovoltage across Au-*h*BN-Graphene.

When the temperature difference is applied across the Au/graphene/*h*-BN heterostructure, the hot electrons in the graphene are ballistically transported to Au by a thermionic emission process, as illustrated in Fig. 1. We assume that the injection of electrons from the graphene into the Au will not change the Fermi level position, which is justified by the very large density of states in the Au. The thermionic emission from graphene is different from Au and can be described by equation^27^ ${J}_{G}={A}_{G}{T}_{h}^{3}\exp \,[-\frac{e{{\rm{\Phi }}}_{Bn}}{{k}_{B}{T}_{h}}]$, where *A*_*G*_ is revised Richardson constant for graphene, *T*_*h*_ is the temperature of graphene, *k*_*B*_ is Boltzmann constant, *e* is elementary charge and Φ_*Bn*_ is the Schottky barrier height at the graphene/*h*-BN interface. The thermionic emission from Au is determined by the equation ${J}_{Au}={A}_{Au}{T}_{c}^{2}\exp \,[-\frac{e{{\rm{\Phi }}}_{Bn}-{V}_{oc}}{{k}_{B}{T}_{c}}]$, where *A*_*Au*_ is Richardson constant for Au, *T*_*c*_ is the temperature of Au, and *V*_*oc*_ is the open voltage generated by the temperature difference across heterostructure. The net electric current across the graphene/*h*-BN/Au heterostructure can be determined by the difference of *J*_*G*_ *and J*_*Au*_. When operating under open circuit conditions, the net electric current is zero, and ${V}_{oc}={{\rm{\Phi }}}_{Bn}(1-\frac{1}{1+\frac{{\rm{\Delta }}T}{{T}_{c}}})+\frac{{k}_{B}{T}_{c}}{e}\,\mathrm{ln}(\frac{{A}_{G}}{{A}_{Au}}\frac{{({\rm{\Delta }}T+{T}_{c})}^{3}}{{T}_{c}^{2}})$. Within the limit of small temperature gradient Δ*T*, the *V*_*oc*_ is linearly proportional to Δ*T*, which is consistent with experiment data in this work as shown in Fig. 6. For, the theoretical model, we use the barrier height of Φ_*Bn*_ = 125 meV and modified Richardson constant for graphene *A*_*G*_ of 0.104 Acm^−2^K^−3^ ^27^. The Richardson constant for gold *A*_*Au*_ of 31.2 Acm^−2^K^−2^ is obtained by using the effective mass of *h*-BN 0.26 as a correction factor multiplied by universal constant *A*_0_ = 120 Acm^−2^K^−2^ ^28^. Note smooth transition between the two temperature scalingT^3^ and T^2^ has been developed^29^. In Fig. 6, the experimental data shows that the thermovoltage depends linearly on the temperature difference across the heterostructure. Our theoretical model also demonstrates a linear dependence between the thermovoltage and temperature gradient, as represented by the red dots symbol in the Fig. 6. Because we have limited information about the Schottky barrier height at the graphene/*h*-BN interface and the effective Richardson constant for the thermionic emission from Au to *h*-BN, we used typical parameters (ϕ_Bn_ = 125 meV and A_Au_ = 31.2 Acm^−2^K^−2^) to qualitatively show the general trend. Here, the linear behavior is in agreement with experiment, and any discrepancy is likely due to inaccuracies in these parameters and/or minor errors in the characterization of the temperature gradient.

In summary, we have developed a technique for measuring the thermoelectric transport across a graphene/*h*-BN/Au heterostructure. A transparent ITO heater is used to enable the temperature of the graphene top electrode to be measured optically using Raman spectroscopy *in situ* under device operating conditions. We apply an AC voltage at a frequency of ω to the ITO heater and measure the thermoelectric voltage across the Au/*h*-BN/graphene heterostructure at a frequency of 2ω using a lock-in amplifier. We are able to separate the actual thermovoltage from the second harmonic voltage originating from the non-linear current-voltage characteristics of Au/*h*-BN/graphene heterostructure by performing the experiment in “heating” and “non-heating” configurations. We observe a Seebeck coefficient of −215 μV/K for this device. This thermoelectric voltage originates from the graphene/*h*-BN interface due to thermionic emission rather than bulk diffusive transport. As such, this should be thought of as an interfacial Seebeck coefficient rather than a Seebeck coefficient of the constituent materials.

## Electronic supplementary material

Supplementary Information

## References

[1] Britnell L (2012). Field-Effect Tunneling Transistor Based on Vertical Graphene Heterostructures. Science.

[2] Das S, Gulotty R, Sumant AV, Roelofs A (2014). All Two-Dimensional, Flexible, Transparent, and Thinnest Thin Film Transistor. Nano Letters.

[3] Roy T (2015). Dual-Gated MoS2/WSe2 van der Waals Tunnel Diodes and Transistors. Acs Nano.

[4] Roy T (2014). Field-Effect Transistors Built from All Two-Dimensional Material Components. Acs Nano.

[5] Furchi MM, Pospischil A, Libisch F, Burgdoerfer J, Mueller T (2014). Photovoltaic Effect in an Electrically Tunable van der Waals Heterojunction. Nano Letters.

[6] Shanmugam M, Jacobs-Gedrim R, Song ES, Yu B (2014). Two-dimensional layered semiconductor/graphene heterostructures for solar photovoltaic applications. Nanoscale.

[7] Withers F (2015). Light-emitting diodes by band-structure engineering in van der Waals heterostructures. Nature Materials.

[8] Liang, S. J., Liu, B., Hu, W., Zhou, K. & Ang, L. K. Thermionic Energy Conversion Based onGraphene van der Waals Heterostructures. *Scientific Reports* 7, doi:10.1038/srep46211 (2017). 10.1038/srep46211 PMC5384245 28387363

[9] Wan, C. *et al*. Flexible n-type thermoelectric materials by organic intercalation of layered transition metal dichalcogenide TiS2. *Nat Mater* **14**, 622–627, 10.1038/nmat4251http://www.nature.com/nmat/journal/v14/n6/abs/nmat4251.html#supplementary-information (2015). 10.1038/nmat4251 25849369

[10] Shakouri A, Bowers JE (1997). Heterostructure integrated thermionic coolers. Applied Physics Letters.

[11] Mahan GD, Sofo JO, Bartkowiak M (1998). Multilayer thermionic refrigerator and generator. Journal of Applied Physics.

[12] Mahan GD, Woods LM (1998). Multilayer thermionic refrigeration. Physical Review Letters.

[13] Chen C-C, Li Z, Shi L, Cronin SB (2015). Thermoelectric transport across graphene/hexagonal boron nitride/graphene heterostructures. Nano Research.

[14] Cai W (2010). Thermal Transport in Suspended and Supported Monolayer Graphene Grown by Chemical Vapor Deposition. Nano Letters.

[15] Chen C-C, Aykol M, Chang C-C, Levi AFJ, Cronin SB (2011). Graphene-Silicon Schottky Diodes. Nano Letters.

[16] Chen, C.-C., Chang, C.-C., Li, Z., Levi, A. F. J. & Cronin, S. B. Gate tunable graphene-silicon Ohmic/Schottky contacts. *Applied Physics Letters* **101**, doi:10.1063/1.4768921 (2012).

[17] Chen, Z., Jang, W., Bao, W., Lau, C. N. & Dames, C. Thermal contact resistance between graphene and silicon dioxide. *Applied Physics Letters* **95**, doi:10.1063/1.3245315 (2009).

[18] Koh YK, Bae M-H, Cahill DG, Pop E (2010). Heat Conduction across Monolayer and Few-Layer Graphenes. Nano Letters.

[19] Mak, K. F., Lui, C. H. & Heinz, T. F. Measurement of the thermal conductance of the graphene/SiO2 interface. *Applied Physics Letters* **97**, doi:10.1063/1.3511537 (2010).

[20] Mao, J. *et al*. Silicon layer intercalation of centimeter-scale, epitaxially grown monolayer graphene on Ru(0001). *Applied Physics Letters* **100**, doi:10.1063/1.3687190 (2012).

[21] Sadeghi MM, Pettes MT, Shi L (2012). Thermal transport in graphene. Solid State Communications.

[22] Schmidt, A. J., Collins, K. C., Minnich, A. J. & Chen, G. Thermal conductance and phonon transmissivity of metal-graphite interfaces. *Journal of Applied Physics* **107**, doi:10.1063/1.3428464 (2010).

[23] Yue Y, Zhang J, Wang X (2011). Micro/Nanoscale Spatial Resolution Temperature Probing for the Interfacial Thermal Characterization of Epitaxial Graphene on 4H-SiC. Small.

[24] Vallabhaneni AK, Singh D, Bao H, Murthy J, Ruan X (2016). Reliability of Raman measurements of thermal conductivity of single-layer graphene due to selective electron-phonon coupling: A first-principles study. Physical Review B.

[25] Sullivan S (2017). Optical Generation and Detection of Local Nonequilibrium Phonons in Suspended Graphene. Nano Letters.

[26] Castellanos-Gomez, A. *et al*. Deterministic transfer of two-dimensional materials by all-dry viscoelastic stamping. *2d Materials* **1**, doi:10.1088/2053-1583/1/1/011002 (2014).

[27] Liang SJ, Ang LK (2015). Electron Thermionic Emission from Graphene and a Thermionic Energy Converter. Phys. Rev. Appl..

[28] Xu YN, Ching WY (1991). Calculation Of Ground-State And Optical-Properties Of Boron Nitrides In The Hexagonal, Cubic, And Wurtzite Structures. Physical Review B.

[29] Ang, Y. & Ang, L. Current-Temperature Scaling for a Schottky Interface with Nonparabolic Energy Dispersion. *Phys. Rev. Appl.* **6**, 034013 (2016).

