# Supplementary material for: Cross-plane Thermoelectric and Thermionic Transport across Au/h-BN/Graphene Heterostructures
Source: Sci Rep. 2017 Oct 26;7:14148. doi: 10.1038/s41598-017-12704-w (PMC5658445; doi:10.1038/s41598-017-12704-w)
Supplement: Supplementary file 1 — Supplementary Information [file 41598_2017_12704_MOESM1_ESM.pdf]

Supplemental Information for

Cross-plane Thermoelectric and Thermionic Transport across Au/*h*-BN/Graphene Heterostructures

Nirakar Poudel[1], Shi-Jun Liang[2], David Choi[3], Bingya Hou[1], Lang Shen[4], Haotian Shi[4], Ang Lay Kee[2], Li Shi[3], and Stephen Cronin[1,5,6]

[1]Ming Hsieh Department of Electrical Engineering, [4]Mork Family Department of Chemical Engineering and Materials Science, [5]Department of Chemistry, [6]Department of Physics and Astronomy, University of Southern California, Los Angeles, CA 90089, USA

[3]Department of Mechanical Engineering and Texas Materials Institute, University of Texas, Austin, Texas 78712, USA

[2]Engineering Product Development (EPD)
Singapore University of Technology and Design (SUTD)

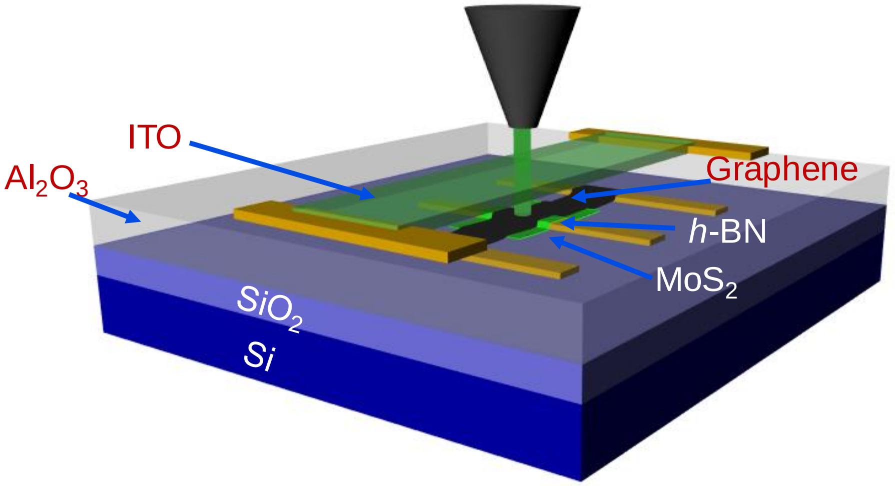

Figure S1. Diagram illustrating the Au/MoS_2/BN/graphene test structure.

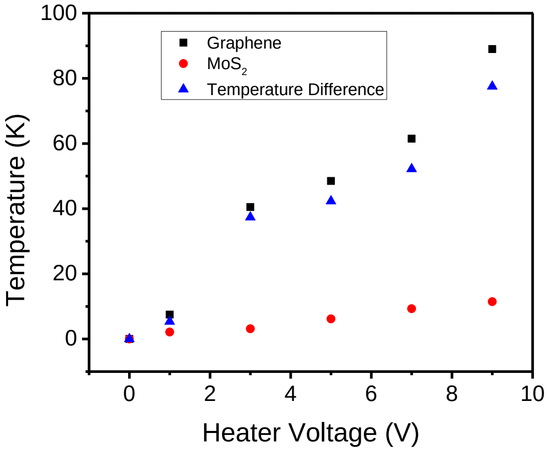

Figure S2. Graphene and MoS_2 temperatures measured by Raman spectroscopy plotted as a function of heater voltage. This data shows that there is negligible heating in the MoS_2 layer and, hence, the underlying gold layer.
